# The Search for an Ideal Temporary Skin Substitute: *AW*BAT

**Published:** 2009-02-12

**Authors:** E. Aubrey Woodroof

**Affiliations:** Aubrey, Inc, 5930 Sea Lion Pl, Carlsbad, CA 92010

## Abstract

**Objective:** The search for an ideal temporary skin substitute is a continuous quest. Without the ability to provide active transport systems powered by adenosine triphosphate or adenosine diphosphate that pump fluid out on demand, all skin substitutes, however effective, would be a compromise. Therefore, the best that any current wound covering design can do is to strive to produce all the other qualities of an ideal skin substitute. Recently developed technology utilized in *AW* BAT attempts to better maximize those attributes. **Methods:** Desirable traits of an ideal skin substitute include adherence, moisture permeability control, infection control, safety, pain management, physical adaptability, transparency, stability, cost-effectiveness, and ease of application and removal. Analysis of the optimization of these traits exemplifies the proficiency of a skin substitute. **Results:** Improvements in porosity and manufacturing methodology intend to increase *AW*BAT's capability over existing products to better fulfill the ideal properties of a skin substitute. **Conclusion:** It is expected that new technological improvements in *AW*BAT will provide improved performance over existing skin substitutes. Increased porosity and continuity of the 3-dimensional silicone-nylon membrane are expected to improve acute adherence and reduce the potential for infection associated with fluid accumulation, and the elimination of cross-linking agents in the collagen application is expected to improve the interaction with fibrin and eliminate the potential for allergic reaction to those agents.

The search for an ideal temporary skin substitute, or bioengineered alternative tissue, for the effective closure of superficial, partial-thickness, and excised full-thickness wounds is continually ongoing. At this point, the best any temporary skin replacement system can do is to maximize the attributes of the ideal skin substitute. For example, since current state-of-the-art bioengineering and biotechnology are not capable of designing active transport systems powered by adenosine triphosphate or adenosine diphosphate into skin substitutes to pump fluid out on demand, any skin substitute, however effective, is a compromise. Biobrane, the first biologically based wound dressing cleared by the FDA^1^ in 1979, was designed with the ideal properties in mind. Since then, many dressings have been introduced, but, to date, none has provided an optimal solution. Recently, principles of biochemistry and bioengineering have been employed to create *AW*BAT, which was cleared by the FDA in February, 2009. *AW*BAT has been designed to provide the clinician with the most advantageous temporary skin substitute.

Biobrane has been proven in multicenter trials to be an effective alternative to fresh and frozen allograft and glutaraldehyde-treated porcine xenograft.^2^,^3^ *AW*BAT, an extension of the design of Biobrane, is intended to better satisfy the desired attributes of an ideal temporary skin substitute.^4^–^8^ Significant design changes to the silicone-nylon membrane include increased porosity of the silicone layer and the continuity of the 3-dimensional nylon structure. Additional changes include the elimination of the cross-linking agents, cyanuric acid and dodecylamine, utilized in Biobrane to covalently bond the porcine type 1 collagen peptide to the silicone-nylon membrane.^9^

## NOMENCLATURE

Alternative terminology^10^ has been proposed for products referred to as “bioengineered skin,” “bioengineered skin substitutes,” “tissue-engineered skin,” and “bioengineered skin equivalents.” The new term, *bioengineered alternative tissue*, applies to products like Biobrane, Integra, TransCyte, Alloderm, Orcell, Oasis, Epicel, GammaGraft, and Laserskin. Composition of these products includes xenogenic material, such as collagen or chondroitin sulfate, and other biologically derived components, living cells and synthetic materials. *AW*BAT (*Advanced Wound* Bioengineered Alternative Tissue) is a precision, porous silicone membrane bonded with a continuous 3-dimensional nylon structure that contains non–cross-linked porcine type 1 collagen peptides.

In this article, the term *skin substitute* is intended to mean a “bioengineered alternative tissue” to skin for temporary coverage of donor sites, meshed autografts, partial-thickness burns, or excised full-thickness wounds until autograft is available for permanent closure.

## IDEAL PROPERTIES OF A TEMPORARY SKIN SUBSTITUTE

The desirable properties of an ideal temporary skin substitute include the maximization of adherence, moisture permeability control, infection control, safety, pain management, physical adaptability, transparency, stability, cost-effectiveness, and ease of application and removal.

### Adherence

Adherence is believed to be the single most important property of a skin substitute.^4^–^6^,^11^ Adherence of bovine collagen membrane cross-linked with glutaraldehyde was shown to be superior to autograft, homograft, and xenograft on full-thickness and split-thickness wounds but inferior to autograft and homograft on granulating wounds.^12^,^13^ In the development of Biobrane, adherence testing using the quantitative model of Tavis et al was performed on various prototypes. In this study, Biobrane's silicone-nylon membrane had a variety of biological compounds covalently bonded to all surfaces; the composites were tested for adherence at 5 and 72 hours. The bound elements included heparin, chondroitin-4-sulfate, egg albumin, collagen (tropocollagen—rat skin), lysine, fibrinogen, hemoglobin, aspartic acid, alanine, glutamic acid, glycine, glycylglycine, human albumin, collagen peptide (porcine type 1) and lecithin. The one combination that exhibited the best acute adherence over the control, which had nothing added, was the silicone-nylon membrane with porcine type 1 collagen peptide.^9^

*AW*BAT, like Biobrane, is a porous silicone-nylon membrane utilizing porcine type 1 collagen peptides. Adherence of *AW*BAT, however, is anticipated to be superior to Biobrane for 2 reasons. First, the collagen in *AW*BAT is not cross-linked to the silicone-nylon membrane as it is in Biobrane; therefore, without steric hindrance it is expected to react more quickly with the fibrin in the wound to achieve clotting and acute adherence, within the 3-dimensional nylon structure. Second, the porosity increase from Biobrane to *AW*BAT, which is 500% to 1000% (Table 1), is expected to reduce fluid accumulation beneath the dressing and minimize fluid pressure that might force the skin substitute from the wound bed.

## Moisture permeability control

A moist wound environment^4^,^14^ is very important to maximize wound healing and minimize infection complications; however, fluid accumulation beneath a skin substitute can compromise adherence and provide a site for endogenous bacteria to proliferate. Therefore, it is important that a dressing simultaneously allow moisture retention, gaseous transfer, and exudate drainage. This is accomplished through a precision porous silicone membrane design.

Gases, such as oxygen, carbon dioxide, and water vapor, diffuse through solid yet thin silicone membranes, which have been successfully used as membrane oxygenators.^15^ The thickness of the silicone membrane for successful transfer of gases, per the Lande-Edwards Membrane Oxygenator, is 0.001 inch or less. Both Biobrane^9^ and *AW*BAT have this similar silicone thickness, allowing for gaseous transmission while maintaining the ability to control water vapor loss.

**Figure 1:**
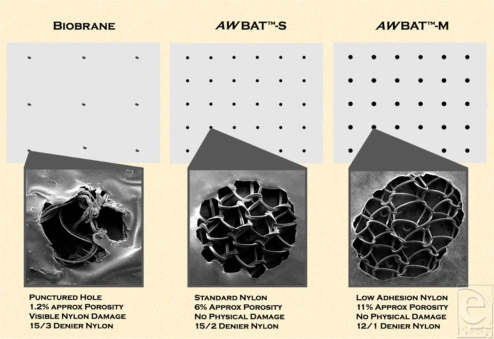
Biobrane and *AW*BAT comparative structure and design: an illustration of the difference in the structure of Biobrane, *AW*BAT-S and *AW*BAT-M with respect to pore density, pore configuration, and nylon structure integrity. The left scanning electron microscope (SEM) image depicts a hole of Biobrane and the destruction of the 3-dimensional nylon structure. The center image depicts a pore of *AW*BAT-S, which, designed as a superficial burn cover, is 500% the porosity of Biobrane. The right image depicts a pore of *AW*BAT-M, which, designed as a meshed autograft cover, is 1000% the porosity of Biobrane. Both *AW*BAT photographs show the 3-dimensional structure of the nylon remaining intact and undamaged.

While a solid silicone membrane is necessary to control water vapor loss, in order to minimize fluid accumulation beneath a skin substitute and allow for exudate removal, wound dressings need to be porous. Biobrane was designed and is manufactured with holes of approximately 1.5 mm diameter punctured through the silicone-nylon membrane at approximately 0.5-inch centers. While this allows for some drainage, complications of fluid accumulation beneath the dressing, leading to suppuration requiring “windowing” and antimicrobial intervention, have been reported.^16^ The patent-pending designs of *AW*BAT enable the placement of precision pores in the silicone membrane without damaging or disrupting the continuous 3-dimensional nylon structure (Fig 1). It is believed that a continuous nylon structure over the entire wound surface will better enable tissue ingrowth and uniform healing with minimal scarring. The pores in *AW*BAT are spaced and sized in a fashion to increase the porosity 500% to 1000% over Biobrane (Table 1), with the intent to further minimize fluid accumulation beneath the skin substitute and the associated complications.

By contrast, Integra's silicone membrane (RTV) was not manufactured with pores; as a result, fluid could accumulate beneath the membrane, which can lead to infection and complications. The manufacturer^17^ has subsequently recommended meshing the membrane to resolve this issue. Heimbach^18^ stated that his preference is to mesh Integra in order to minimize fluid accumulation.

## Infection control

The best defense against infection is a wound covering that sufficiently acts as a bacterial barrier, which the thickness of the silicone membrane in Biobrane and *AW*BAT provides, and the proper preparation of the wound. Improperly prepared and/or contaminated wounds are not amenable to skin substitutes such as fresh or frozen human cadaver allograft, porcine xenograft, Integra, Biobrane, and *AW*BAT.^16^ It is believed that when dressings such as these are applied to clean, surgically debrided, or excised hemostatic wounds, antimicrobial impregnation is not usually necessary.

Some clinicians have recently looked to antimicrobial activity as a safeguard against infection, and many recently developed dressings contain silver ion as an antimicrobial agent. While Biobrane, at approximately 1.2% porosity, was not designed specifically to address the potential desire for antimicrobial permeability, *AW*BAT is more accommodating. *AW*BAT-S, designed with 6% porosity, is 500% more permeable to water-soluble antimicrobials such as mafenide acetate (Sulfamylon). *AW*BAT-D and *AW*BAT-M, with 12% porosity, are 1000% more permeable. Diffusion of antimicrobials is directly related to the concentration gradient of the antimicrobial across the skin substitute and the skin substitute's porosity. The ability to treat endogenous microbes without removal of the skin substitute by diffusing antimicrobials through the skin substitute is highly desirable. It is believed that use of a water-soluble antimicrobial over *AW*BAT in “borderline” contaminated wounds could control the proliferation of endogenous bacteria without necessitating removal of the dressing.

## Physical adaptability

Stretchability, flexibility, conformability, and stability of a skin substitute are important to the clinician,^9^ particularly when the wound is irregularly contoured. The stretchability and flexibility of the dressing are critically important during rehabilitation when movement is desired. The nylon component and the thinness of the silicone membrane in Biobrane and *AW*BAT allows for these properties. Many wound dressings lacking these qualities become stiff, inflexible, or “cast-like.” Other dressings can shrink or become degraded by wound proteases.

## Safety

Dressings should be sterile, hypoallergenic and nontoxic. Some skin substitutes, such as fresh and frozen allograft, cannot guarantee sterility. Cadaver allograft has the potential to transmit acquired immunodeficiency syndrome and hepatitis. As both glutaraldehyde and formaldehyde are useful as sterilizing agents and reduce the antigenicity of biological tissues,^19^ porcine xenograft has been treated with glutaraldehyde to improve its shelf life and sterility safety margins; however, in the cross-linking process, the physical properties of the product, such as adherence, have been compromised. Biobrane has proven to be safe, with the exception of rare allergic reactions.^20^

Production of *AW*BAT is controlled to maximize safety and sterility (Table 2), including collagen application methods that eliminate the cross-linking agents used with Biobrane—cyanuric acid and dodecylamine.

Punctate scarring is another rare complication reported with Biobrane.^21^ While the cause is unknown, the scarring is aligned with the holes of Biobrane where the 3-dimensional nylon structure is disrupted. The continuity of nylon at *AW*BAT's pores is intended to mitigate this potential.

## Pain management

Pain reduction and patient comfort are desirable in wound care. Occlusive dressings that appropriately cover exposed nerve endings and provide moist wound healing environments characteristically reduce pain. They contrast sharply with nonocclusive dressings such as scarlet red, Xeroform, and fine mesh gauze in their ability to ease pain.^4^ Barret et al^22^ report that Biobrane shows the significant benefits of being less painful with fewer dressing changes over conventional antimicrobial treatments for second-degree burns in children. *AW*BAT, like Biobrane, is a semiocclusive temporary skin substitute and is expected to reduce pain, provide patient comfort, and enable quicker rehabilitation and ambulation.

## Transparency

*AW*BAT, like Biobrane, is transparent to allow the clinician to observe the healing process at the wound surface. The ability to detect suppuration and implement immediate corrective action such as antimicrobial therapy is a distinct advantage over products lacking transparency.

## Stability

Long shelf life at room temperature is highly desirable.^4^ Storage requirements for allograft, TransCyte, and other skin substitutes at refrigerated or subzero temperatures translate into additional expense and inconvenience. *AW*BAT, like Biobrane, is designed to be sterile and stable at room temperature for 3 years in an unaltered package.

## Cost-effectiveness

The ultimate cost of any skin substitute includes not only the product but also the length and complexity of patient care, including the expenses of dressing changes, complications, and hospital stay. *AW*BAT, like Biobrane, is designed to remain in place on superficial burns, donor sites, and meshed autografts for the life of the wound. It is also designed to mitigate the potential for infection. Biobrane has proven to be cost-effective on superficial burns compared with silver sulfadiazine (Silvadene) wound management, and some insurance companies consider Biobrane medically necessary for the management of burns.^3^,^22^,^23^

## Ease of application and removal

The ability to easily secure a skin substitute with staples, sutures, or steri-strips and easily remove it in whole pieces when necessary is desirable. *AW*BAT, like Biobrane, has a stretchable, conformable silicone-nylon component that can be easily attached by the clinician. The silicone-nylon membrane also has sufficient strength to enable removal without tearing.

## CLINICAL EVALUATIONS

Numerous clinicians have expressed an interest in *AW*BAT and also in clinically evaluating its efficacy. A pilot study will evaluate *AW*BAT-S and *AW*BAT-D in a donor site model. Other multicenter studies will examine *AW*BAT-S against Biobrane on clean, acute superficial burns and *AW*BAT-D against Xeroform, Glucan II, and MepilexAg on donor sites.

## WOUND HEALING ENHANCEMENT

In addition to the properties listed above, the optimal temporary skin substitute would also enhance the body's ability to heal itself. TransCyte, known for its ability to accelerate healing,^24^ used Biobrane as the scaffold on which newborn human fibroblast cells were cultured. As the fibroblasts proliferated within Biobrane's nylon mesh during the manufacturing process, they secreted human dermal collagen, matrix proteins, and growth factors. The product was then frozen and required storage at -70°F. While no cellular metabolic activity remained after the freezing process, the tissue matrix and bound growth factors were left intact. Although seemingly successful, it is believed that TransCyte was discontinued because it proved too expensive to manufacture and maintain.

**Figure 2:**
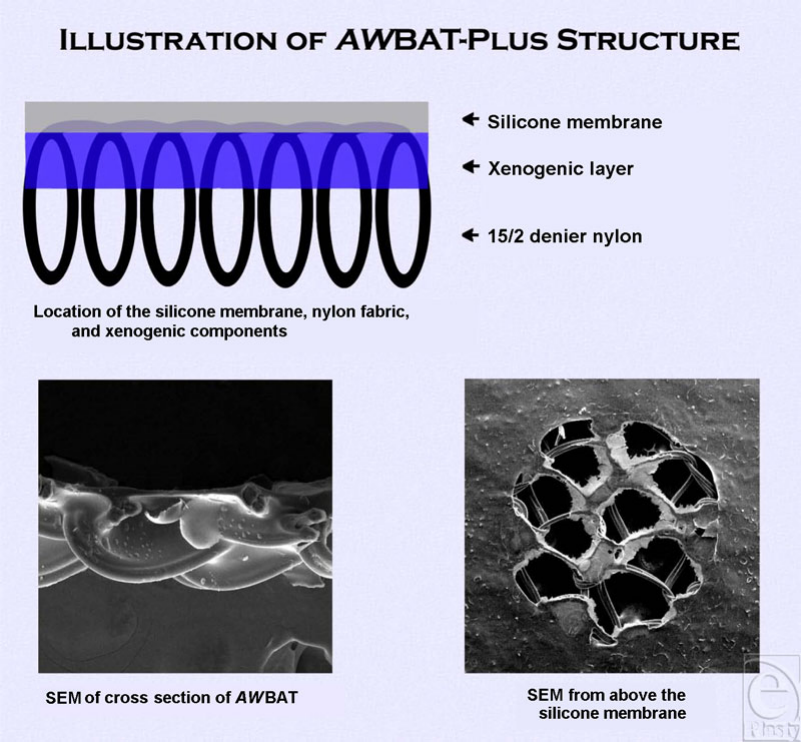
*AW*BAT-Plus.

As the search for an ideal temporary skin substitute is continually ongoing, the next generation of *AW*BAT is currently under development. *AW*BAT-Plus, with patent-pending design, is being created with the intent not only to capitalize on maximizing the ideal properties discussed herein but also to cost-effectively enhance wound healing. *AW*BAT-Plus is similar to *AW*BAT, utilizing the same precision porous silicone-nylon membrane, except it contains 6 xenogenic components (Fig 2). In addition to a substantial increase in the volume of porcine type I collagen peptides, it includes chondroitin-4-sulfate, chondroitin-6-sulfate, vitamin E, vitamin C, and Immuno-10 (a highly purified fraction of *Aloe*).

## CONCLUSION

While the search for the optimal skin substitute will continue, it is believed that *AW*BAT, by design, beneficially expands upon the attributes of existing temporary skin substitutes to better fulfill the ideal properties of a bioengineered alternative tissue. The increased porosity and continuity of the 3-dimensional silicone-nylon membrane is expected to improve acute adherence and to reduce the potential for infection associated with fluid accumulation. The elimination of cross-linking agents in the collagen application is expected to improve the interaction with fibrin and to eliminate the potential for allergic reaction to those agents. The retained benefits of pain management, physical adaptability, transparency, stability, cost-effectiveness, and ease of application and removal further define its advantages. *AW*BAT-Plus, with the additional biological components, is projected to further improve upon *AW*BAT's benefits by enhancing wound healing.

## Figures and Tables

**Table 1:** Similarities and differences of the Biobrane and AWBAT designs

Attributes	Biobrane	*AW*BAT-S	*AW*BAT-D	*AW*BAT-M
Intended use	Superficial burns, donor sites, excised wounds (Biobrane); meshed autografts (Biobrane-L)	Superficial burns	Donor sites	Meshed autografts
Design objective	15/3 (Biobrane) or 12/1 (Biobrane-L) denier nylon—adherence strong (Biobrane) or light (Biobrane-L), 98.8% occlusive, single porosity (1.2%)	15/2 denier nylon—strong adherence, 94% occlusive, porosity 6%	15/2 denier nylon—strong adherence, 88% occlusive, porosity 12%	12/1 denier nylon—weak acute and secondary adherence, 89% occlusive, porosity 11%
Silicone component	Medical grade silicone	Medical grade silicone	Medical grade silicone	Medical grade silicone
Collagen peptide component	Porcine type 1 collagen peptide (covalently bound)	Porcine type 1 collagen peptide (mobile)	Porcine type 1 collagen peptide (mobile)	Porcine type 1 collagen peptide (mobile)
Cross-linking agents used	Dodecylamine, cyanuric chloride^4^	None used	None used	None used
Sterilizing method	Autoclave	25 kGy E-beam irradiation	25 kGy E-beam irradiation	25 kGy E-beam irradiation
Shelf life, y	3	3	3	3

**Table 2:** AWBAT safety and sterility results from North American Science Associates, Inc

Test	Scope	Conclusion
ISO muscle implantation study	2-wk evaluation for evidence of irritation or toxicity	Macroscopic reaction was not significant as compared with the negative control implant; microscopically classified as a nonirritant
ISO systemic toxicity study	0.9% sodium chloride USP solution and alcohol in saline 1:20 solution extracts evaluated for systemic toxicity	No mortality or evidence of systemic toxicity from the extracts
ISO systemic toxicity study	Sesame oil extracts evaluated for systemic toxicity	No mortality or evidence of systemic toxicity from the extracts
ISO intracutaneous study	0.9% sodium chloride solution and sesame oil extracts evaluated for intracutaneous reactivity	No erythema and no edema from the SC extract and no to well-defined erythema and edema from the SC extracts
ASTM hemolysis	Test article and extract in calcium- and magnesium-free phosphate-buffered saline evaluated for in vitro red blood cell hemolysis	The direct contact of the test article was nonhemolytic, and the test article extract was nonhemolytic
ISO maximization sensitization study	0.9% sodium chloride solution and sesame oil extracts evaluated for the potential for delayed dermal-contact sensitization	The SC and SO test article extracts showed no evidence of causing delayed dermal-contact sensitization
Cytotoxicity study using the ISO elution method	Single strength minimum essential medium supplemented with 5% serum and 2% antibiotics studied for biocompatibility	Test article showed no evidence of causing cell lysis or toxicity
E-beam radiation sterilization	Establish radiation dose and validate the effectiveness of E-beam radiation for sterilization	Reaching minimum recommended dose, the overall adjusted bioburden was 31.7 CFU's per device, less than the maximum bioburden of 1000 CFU allowed by ISO 11137–2

ISO, International Organization for Standardization; USP, United States Pharmacopeia; ASTM, American Society for Testing and Materials; SC, Sodium Chloride; SO, Seasame Oil: CFU, colony-forming unit.
